# Pharmacist medication instructions are associated with continued medication self-management in older adults: a retrospective observational study

**DOI:** 10.1186/s40780-021-00194-y

**Published:** 2021-03-03

**Authors:** Eiji Kose, Hidetatsu Endo, Hiroko Hori, Shingo Hosono, Chiaki Kawamura, Yuta Kodama, Takashi Yamazaki, Nobuhiro Yasuno

**Affiliations:** 1 Department of Pharmacy, Teikyo University School of Medicine University Hospital, 2-11-1 Kaga, Itabashi-ku, Tokyo, 173-8606 Japan; 2 Department of Pharmacy, Ogaki Tokushukai Hospital, 6-85-1 Hayashi-chou, Ogaki, Gifu, 503-0015 Japan; 3 Laboratory of Hospital Pharmacy, School of Pharmacy, Teikyo University, 2-11-1 Kaga, Itabashi-ku, Tokyo, 173-8606 Japan

**Keywords:** Convalescent rehabilitation ward, Medication self-management, Older adults, Pharmacist medication instructions

## Abstract

**Background:**

Various factors are related to self-management of medication. However, few reports comprehensively examine the factors related to patients, medication levels, and other factors related to the recuperative environment, such as family support. The aim of this study was to investigate factors affecting the continuation of medication self-management among hospitalized older adults receiving convalescent rehabilitation.

**Methods:**

We conducted a retrospective observational study with 274 consecutive patients newly admitted to the convalescent rehabilitation wards at a single hospital in Japan between January 2017 and May 2018. Participants who were assessed for their ability to take their medication using the Japanese Regimen Adherence Capacity Tests, were deemed to be self-manageable, and were able to successfully continue to self-manage their medication from admission to discharge were categorized as the “continuation group,” and those who were not able to continue were categorized as the “non-continuation group.” We analyzed the groups’ demographic data, laboratory data, and Functional Independence Measure. The primary outcome was the continuation of medication self-management from admission to discharge.

**Results:**

After enrollment, 134 patients (median age 82 years; 62.7% women) were included in the final analysis. Some 60.4% of eligible patients were able to maintain medication self-management during their hospitalization. The multiple logistic regression analysis for the continuation of medication self-management during hospitalization after adjusting for confounding factors revealed that pharmacist medication instructions were independently and positively correlated with successful continuation of medication self-management (odds ratio: 1.378; 95% confidence interval 1.085–1.831; *p* = 0.0076).

**Conclusion:**

Successful continuation of medication self-management is associated with pharmacist medication instructions among hospitalized older adults undergoing rehabilitation.

**Trail registration:**

The Ethics Committee’s registration number is “TGE01216–066”.

## Background

The primary purpose of the convalescent rehabilitation ward is to provide intensive rehabilitation for patients with stroke, femur fracture, and hospital-associated deconditioning. The second aim is to improve and maintain ADL to help patients return home. The convalescent rehabilitation ward is characterized by a long hospital stay and a significant number of elderly patients. Thus, not only the direct cause of their hospitalization, but also their underlying medical condition prior to admission makes many elderly patients prone to polypharmacy due to the high number of medications they take. A previous report had shown that 33% of patients in the convalescent rehabilitation ward were found to have polypharmacy [1]. Polypharmacy is a known cause of poor medication adherence [2]. Approximately 34% of patients with stroke failed to maintain their medication after 1 year of discharge [3]. Poor medication adherence has a negative impact on the treatment and control of disease and also affects the occurrence of adverse effects due to medications.

Many patients in the convalescent rehabilitation ward recuperate at home after discharge; therefore, it is important to improve their medication knowledge during hospitalization to maintain long-term medication self-management. Previously, patient-level barriers associated with medication self-management included the ability to ensure self-care, interest attached to a stroke event, and knowledge of stroke and medication. Medication-level barriers included beliefs about medication and beliefs about how pills work, medication routines, changing medications, regimen complexity, and burden of treatment [4]. Some studies have reported that patients seek psychological and emotional support in the process of self-management and that they also sometimes need family support; the influence of the recuperative environment must be considered in the context of medication self-management [5, 6]. Although various factors are related to self-management of medication, few reports have comprehensively examined factors related to patients, medication levels, and other factors related to the recuperative environment, such as family support.

In this study, we examined the factors affecting the continuation of medication self-management in elderly patients in the convalescent rehabilitation ward, taking into account factors related to patients and their medication levels as well as factors in the recuperative environment, such as family support.

## Methods

### Study design and participants

A retrospective observational study was performed on 274 consecutive participants admitted to the convalescence rehabilitation ward at Ogaki Tokushukai Hospital between January 2017 and May 2018. The inclusion criterion was age ≥ 65 years. Participants who were self-managing their medication at admission, with severe communication difficulties, with severe psychiatric disorders, with severe higher brain dysfunction or cognitive decline, who refused to self-manage their medication, who had no prescription medication, who were institutional/family-managed, or who were health care providers, readmitted participants, or who were clearly unable to self-manage for other reasons were excluded.

### Investigation items

Data on the participants’ basic information were collected from their medical records at admission and discharge, as appropriate. This information included their age, sex, length of stay, body weight, BMI, primary diagnosis, comorbidities, presence of family cooperation, HDS-R, upper limb paralysis, higher brain dysfunction, vision loss, number of medications at admission, medication instruction, number of medication instructions, and FIM.

Laboratory data were also collected from medical records at admission and discharge. These included albumin and CRP levels.

### Assessment indications

Indicators of ADL such as FIM and the Barthel index are used for evaluation during the recovery period [7]. In particular, the reliability of FIM is confirmed in a meta-analysis of 11 studies [8]. Therefore, we used FIM as the ADL measurement. The FIM score, which includes 13 lower-order items regarding FIM-M and 5 lower-order items regarding FIM-C, is one of the most common measures of ADL [8]. Each item is scored on a scale of 1 point (total assistance) to 7 points (complete independence). The FIM-T score therefore ranges from 18 to 126 points. FIM scores were determined at admission and discharge by a multidisciplinary rehabilitation team, including a rehabilitation physician, registered dietitian, nurse, physical therapist, occupational therapist, speech language-hearing therapist, and pharmacist. Based on clinical judgment, appropriate rehabilitation was offered to all participants, regardless of their FIM score, stroke severity, or length of stay.

In this study, we used the J-RACT to assess the patient’s ability to take medication. The J-RACT is a medication assessment test to evaluate the ability of elderly people with disease in Japan to take medication and to provide them with appropriate medication instructions. J-RACT was originally developed based on the report by Fitten et al. [2]. However, the method and content of J-RACT were completely redesigned to conform to Japanese medication. J-RACT has been used to examine the ability of prevalent older patients with chronic cardiovascular disease to take medication, mainly in outpatient and inpatient settings [9]. The J-RACT consists of 4 simple questionnaires for the elderly on (1) visual and auditory acuity; (2) ability to understand medication (understanding of dosage and administration); (3) ability to work with medication (manual dexterity); and (4) ability to manage medication (long-term management at home) [9]. The ability to manage medications (long-term management at home) was assessed by the RCS. The RCS is a medication management assessment scale that uses 5 medications with various dosing methods and medication bags and asks questions about the 5 dosing methods using an interview method. The RCS is a 10-point scale divided into 4 levels based on scores: normal ability (10 points), caution required (9, 8 points), training required (7, 6 points), and assistance required (5 points or less) [10]. A multidisciplinary team, including physicians, pharmacists, and nurses with long experience in convalescent rehabilitation, comprehensively assessed items (1) to (4) and determined whether the patient could self-manage his or her medication. Participants who were assessed for their ability to take their medication using the J-RACT, were deemed self-manageable, and were able to successfully continue to manage their medication from admission to discharge were categorized as the “continuation group,” and those who were not able to continue were categorized as the “non-continuation group.”

### Outcome measurement

The primary outcome of this study was the continuation of successful medication self-management from admission to discharge.

### Statistical analysis

All statistical analyses were performed using JMP Pro (Version 13, SAS Institute, Cary, NC, USA). Data with a normal distribution were described as mean ± standard deviation. If not normally distributed, data were described as median (interquartile range 25th–75th percentiles). A *p*-value < 0.05 was considered statistically significant.

Student’s *t*-test, the Mann–Whitney U test, and χ^2^ test were used to analyze the differences between groups. A multiple logistic regression analysis was used to analyze the factors influencing the continuation of medication self-management during hospitalization. Factors found to be significantly associated in the univariate analysis of continued medication self-management (age, FIM-C at admission, number of medication instructions, HDS-R, albumin level at admission, hypertension, and dyslipidemia) were used as explanatory variables in the multiple logistic regression analysis. Only 1 variable, which was more reasonable from a medical and pharmacological point of view, was included in the multiple logistic regression analysis if there was a strong internal correlation between the explanatory variables (*p* < 0.01). We drew a ROC curve to assess the cut-off value for the number of episodes of medication instruction associated with continued medication self-management. We calculated sensitivity, specificity, accuracy, true positive, true negative, false positive, false negative, F1 value, and MCC [11] to further assess the number of episodes of medication instruction that predicts the continued medication self-management.

## Results

### Study populations

This retrospective observational study with 274 consecutive participants excluded 23 patients who were self-managing their medication at admission, 1 patient with severe communication difficulties, 6 patients with severe psychiatric disorders, 70 patients with severe higher brain dysfunction or cognitive decline, 16 patients who refused to self-manage their medication, 2 patients with no prescription medication, 9 patients under institutional or family management, 1 health care provider, 2 patients who were readmitted to the hospital, and 10 patients who were clearly unable to self-manage for other reasons. Ultimately, 134 participants with a median age of 82 years (interquartile range 76–85 years; 84 women) were included, of whom 10 (7.5%) had cerebral infarction, 7 (5.2%) had intracerebral hemorrhage, 2 (1.5%) had subarachnoid hemorrhage, 104 (77.6%) had osteoporosis-related diseases, 1 (0.7%) had hospital-associated deconditioning, and 10 (7.5%) had other conditions.

### Descriptive and univariate analyses

Table 1 shows the demographic characteristics of the patients based on the presence (*n* = 81) and absence (*n* = 53) of continued medication self-management during hospitalization. BMI at admission, the diagnosis of hypertension and dyslipidemia, HDS-R, the number of medication instructions, FIM-T, FIM-C at admission, and FIM-T, FIM-M, and FIM-C at discharge were significantly higher in the continuation group compared with the non-continuation group. Conversely, age was significantly higher in the non-continuation group compared with the continuation group. There was no significant difference between the 2 groups in any of the other items.

**Table 1:** Baseline of demographic characteristics and laboratory data

Characteristic	All patients (*n* = 134)	Continuation group (*n* = 81)	Non-continuation group (*n* = 53)	*p* value
Age (y)	82 (76–85)	80 (73–84)	83 (80.5–88)	0.0013^†^
Gender *n*, (%)				0.4166^§^
Male	50 (37.3)	28 (34.6)	22 (41.5)	
Female	84 (62.7)	53 (65.4)	31 (58.5)	
Length of stay (d)	42 (29–55.8)	40 (28.5–58.5)	45 (29–54)	0.5571^†^
Body weight at admission (kg)	50 (44–60)	51 (43.1–63.9)	48.2 (42–56.8)	0.0502^†^
BMI at admission (kg/m^2^)	21.4 (18.8–24.1)	22.1 (19.2–25.1)	20.7 (17.8–23.2)	0.0115^†^
Primary diagnosis *n*, (%)				0.6419^§^
Cerebral infraction	10 (7.5)	6 (7.4)	4 (7.6)	
Intracerebral hemorrhage	7 (5.2)	3 (3.7)	4 (7.6)	
Subarachnoid hemorrhage	2 (1.5)	2 (2.5)	0 (0)	
Osteoporosis–related disease	104 (77.6)	64 (79.0)	40 (75.4)	
Hospital-associated deconditioning	1 (0.7)	1 (1.2)	0 (0)	
Others	10 (7.5)	5 (6.2)	5 (9.4)	
Comorbid conditions *n*, (%)				
Cardiac disease	18 (13.3)	11 (13.6)	7 (13.2)	0.9507^§^
Diabetes mellitus	30 (22.2)	15 (18.5)	15 (28.3)	0.1840^§^
Hypertension	87 (64.4)	58 (71.6)	29 (54.7)	0.0452^§^
Dyslipidemia	40 (29.6)	31 (38.3)	9 (17)	0.0085^§^
Dementia	2 (1.5)	0 (0)	2 (3.8)	0.0782^§^
Epilepsy	3 (2.2)	1 (1.2)	2 (3.8)	0.3313^§^
Parkinson’s disease	2 (1.5)	1 (1.2)	1 (1.9)	0.7608^§^
Family cooperation *n*, (%)	117 (87.3)	72 (88.9)	45 (84.9)	0.4981^§^
HDS-R	25 (21–28)	26 (23–28)	23 (19–26)	0.0011^§^
Upper limb paralysis *n*, (%)	22 (16.3)	12 (14.8)	10 (18.9)	0.5357^§^
Higher brain dysfunction *n*, (%)	24 (17.9)	9 (11.1)	15 (28.3)	
Vision loss *n*, (%)	14 (10.4)	9 (11.1)	5 (9.4)	0.7563^§^
No. of medications at admission	6.7 ± 3.5	6.8 ± 3.2	6.8 ± 3.8	0.8388^§^
medication instruction *n*, (%)	134 (100)	81 (100)	53 (100)	0.2146^§^
No. of medication instructions	6 (4–9)	7 (5–10)	5 (2.5–7)	<.0001^†^
FIM at admission (points)				
FIM–M	50 (33–60)	52 (37.5–59)	46 (29.5–63.6)	0.1497^†^
FIM–C	30 (26–35)	32 (29–35)	28 (21.5–33)	0.0011^†^
FIM–T	79 (63–92)	82 (69.5–93)	73 (57.5–91.5)	0.0166^†^
FIM at discharge (points)				
FIM–M	82 (74–86)	83 (76.5–86)	78 (67–85)	0.0084^†^
FIM–C	34 (30–35)	35 (32–35)	30 (26–34.5)	<.0001^†^
FIM–T	114 (103.5–119.5)	117 (108.3–121)	109 (93.5–115.5)	0.0004^†^
Clinical laboratory data at admission				
Alb (g/dL)	3.4 ± 0 .6	3.5 ± 0.5	3.2 ± 0.7	0.0097^‡^
CRP (mg/dL)	0.46 (0.19–1.30)	0.41 (0.11–1.15)	0.68 (0.27–1.39)	0.2354^†^

*Abbreviations: Alb* Albumin, *BMI* Body mass index, *CRP* C-reactive protein, *FIM* Functional Independence Measure, *HDS-R* Hasegawa dementia rating scale-revised

†: Mann–Whitney *U* test, ‡: *t*–test, §: Chi–square test

Regarding clinical laboratory data, albumin level at admission was significantly higher in the continuation group compared with the non-continuation group. There was no significant difference in CRP on admission between the 2 groups.

### Multiple logistic regression analysis

The multiple logistic regression analysis for the continuation of medication self-management during hospitalization after adjusting for potential confounders, including age, FIM-C at admission, the number of medication instructions, HDS-R, albumin level at admission, hypertension, and dyslipidemia is shown in Table 2. There was no multicollinearity between the variables. The multiple logistic regression showed that the number of medication instructions was independently and positively associated with the continuation of medication self-management (odds ratio [OR] 1.378; 95% confidence interval [CI] 1.085–1.831; *p* = 0.0076), age (OR 0.888; 95% CI 0.780–0.993; *p* = 0.0363), FIM-C at admission (OR 1.167; 95% CI 1.025–1.353; *p* = 0.0175), and hypertension (OR 5.902; 95% CI 1.485–29.500; *p* = 0.0106) (Table 2).

**Table 2:** Multiple logistic regression analysis for continuation of self-management of medications

Variable	Adjusted	95% CI (Lower–Upper)	*p* value
	Odds ratio		
Age	0.888	0.780–0.993	0.0363
FIM–C at admission	1.167	1.025–1.353	0.0175
No. of medication instructions	1.378	1.085–1.831	0.0076
HDS-R	1.057	0.889–1.275	0.5348
Alb at admission	0.552	0.172–1.660	0.2844
Hypertension	5.902	1.485–29.500	0.0106
Dyslipidemia	3.834	0.774–25.224	0.1021

*Abbreviations: Alb* Albumin, *BMI* Body mass index, *CI* Confidence interval, *FIM–C* Functional Independence Measure–cognitive, *HDS-R* Hasegawa dementia rating scale-revised

### Cut-off value for the necessary number of episodes of medication instruction

**Fig. 1:**
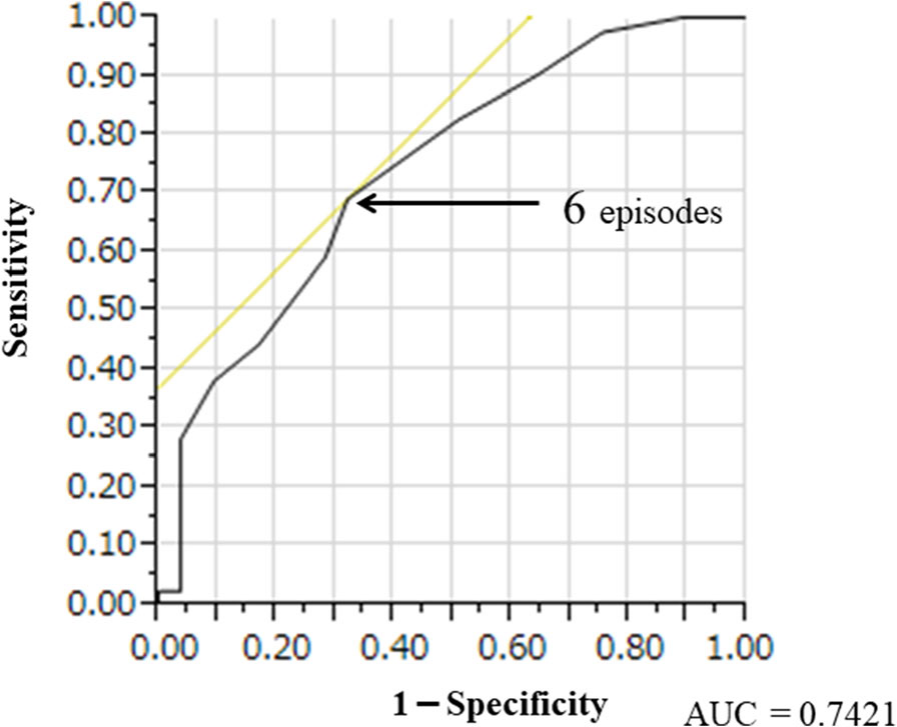
A Cut-off value for the necessary number of episodes of medication instruction for continued medication self-management due to ROC curve analysis

The result of the ROC curve analysis is shown in Fig. 1. Table 3 shows the predictive ability of the predictive number of episodes of medication instruction for continued medication self-management. A cut-off value ≥6 episodes for ROC curve analysis corresponded to 69.1, 67.9, 77.9%, 0.73, and 0.36 sensitivity, specificity, accuracy, F1 value, and MCC, respectively. Meanwhile, the continued medication self-management cut-off value ≥5 episodes had larger values of sensitivity, specificity, F1 value, and MCC, respectively compared to ≥6 or ≥ 7 episodes. Thus, we determined that the cutoff value was ≥5 episodes.

**Table 3:** The verification of the cutoff value for the number of episodes of medication instruction

	TP	FP	TN	FN	Sensitivity	Specificity	Accuracy	F1 value	MCC
≥7 episodes	21	9	28	10	0.677	0.757	0.721	0.689	0.436
≥6 episodes	56	17	36	25	0.691	0.679	0.779	0.727	0.364
≥5 episodes	36	6	16	10	0.783	0.727	0.765	0.818	0.491

*Abbreviations: FN* False Negative, *FP* False Positive, *TN* True Negative, *TP* True Positive, *MCC* Matthews Correlation Coefficient

## Discussion

This study demonstrated that the number of medication instructions by pharmacists were significantly associated with successful continued medication self-management. In other words, pharmacists were found to positively affect patients’ continued medication self-management when they provided medication instructions. Second, the cut-off value for medication instruction episodes required by the pharmacist for continued medication self-management was 5. In other words, when pharmacists repeatedly (5 times) instructed patients on the efficacy and dosage of medications, they were able to maintain successful medication self-management during their hospitalization.

Most of the patients admitted to the convalescent rehabilitation ward are elderly. Therefore, many patients have underlying medical conditions that predate their admission, in addition to the direct cause of their admission. Thus, these patients require long-term adherence to a medication regimen. However, patients admitted to the convalescent rehabilitation ward are often unable to continue their medication self-management and find it difficult to continue taking their medication due to cognitive decline, higher brain dysfunction, and a lack of disease or drug knowledge. Furthermore, from the perspective of rehabilitation pharmacotherapy [12], ADLs may be reduced due to the development of adverse drug events [13–16], resulting in the inability to continued medication self-management. Discontinuing medication at the patient’s own discretion, such as stopping antihypertensive medication, can worsen symptoms and delay their discharge from the hospital. The ability of patients to self-manage their medication and adherence is therefore important to maintaining their ADL.

Medication management during hospitalization has been reported to affect medication adherence after discharge [17]. Sendt et al. had reported that positive attitudes toward medication therapy and disease awareness lead to improved medication self-management and better adherence [18]. In the convalescent rehabilitation ward, due to the nature of the particular condition, such as cognitive decline, higher cerebral dysfunction, or upper extremity paralysis, nurses frequently administer medications to patients for certainty and safety reasons. The nurse gives medication to the patient in the correct dosage and confirms that he or she is taking the medication. Compliance is maintained throughout the hospital stay because it is “managed by the nurses,” so to speak, by simply giving the medication on the spot. However, patients are unlikely to be fully aware of their medications if they are taking them at a set time [17], since patients will no longer have to worry about the dosage and administration of their medications, which is unlikely to improve adherence. It has also been reported that poor adherence is related to the occurrence of adverse effects, the number of medications, and the number of doses [19]. Patients with cognitive impairment are more likely to fall and have other problems than patients with normal cognitive function due to inadequate drug knowledge and understanding of dosage and administration [20]. In the present study, after adjusting for confounding factors such as the number of medications and cognitive functioning, we found that the number of medication instructions was associated with successful continued medication self-management. Therefore, pharmacists should strive to help patients understand the significance of their own medications and repeatedly instruct them to manage their own medication by themselves. We believe that allowing patients to manage their own medications during their hospitalization can improve their willingness to take their medications and help them return to society. Patient supervision by pharmacists is effective in improving clinical laboratory values and medication compliance [21].

The pharmacists in this study used a drug information form to carefully explain the significance and typical adverse effects of each drug in plain language to improve the patients’ drug knowledge. The cut-off value for episodes of medication instruction associated with continued medication self-management was 5. As a rule, the interval for medication instruction was once a week. However, patients were instructed every time there was a change in medications or dosage and administration or the patient’s condition deteriorated. For patients with polypharmacy, we suggested to the physician to reduce dosage or number of medications to prevent the occurrence of adverse effects. We have worked to improve adherence by bundling medications into a 1-dose package, if possible, so that self-management can continue. Medication instructions help patients to acquire correct knowledge about medications and to understand the importance and significance of proper medication use. It is presumed that this instruction reduces anxiety about taking medications and enables patients to continue self-management.

FIM-C on admission and aging were also associated with continued medication self-management. For FIM-C at admission, we demonstrated that higher ADLs related to cognition at admission positively affected continued medication self-management. A previous report showed that older adults with low cognitive functioning have lower medication self-management behaviors, which has been shown to contribute to poor medication compliance [22, 23]. The results of this study differed from those previously reported in that cognitive ADL was associated with medication self-management rather than cognitive function. However, the results of the present study supported previous reports, given cognitive scales such as the Mini-Mental State Examination and the FIM-C have been shown to be significantly correlated [24].

Aging has a negative impact on continued medication self-management. Given aging is associated with decreased medication adherence caused by factors such as cognitive decline and dysphagia [25], it can be an important factor when considering continued medication self-management. A previous study reported a negative correlation between aging and medication self-management [26]; the results of the present study support the previous report.

Hypertension was also associated with continued medication self-management. Many patients with hypertension aim to actively make lifestyle modifications on their own to control blood pressure. Therefore, it is necessary for them to manage their daily activities. In addition, medical professionals routinely provide guidance to hypertensive patients on how to improve their lifestyle. Thus, it was considered that hypertensive patients were generally more aware of self-management and medication self-management, which is considered to be part of their daily lives.

Our study has several limitations. First, because this was a retrospective observational study, a causal relationship between the continuation of medication self-management and medication instructions by a pharmacist could not be established. Therefore, a multicenter prospective study is needed to generalize this study. Second, the lesion site is associated with the self-management of patients with cerebrovascular disease [27]; however, the lesion site in cerebrovascular disease was not considered. Third, the intervals between medication instructions have not been fully investigated. However, at this facility, the highest numbers of medication instructions were only two to three times per week and never five consecutive times. Fourth, the impact of one-dose package on medication management was not considered. Finally, the contents of rehabilitation were not considered.

## Conclusions

Our findings revealed that pharmacist medication instructions might be associated with continued medication self-management during hospitalization. Our findings will be useful in the convalescent rehabilitation ward, where many patients live in their home after discharge. Future studies should examine whether pharmacist medication instructions lead to successful medication self-management.

## Data Availability

All data generated or analyzed during this study are included in this published article.

## Abbreviations

ADL: activities of daily living
BMI: body mass index
CRP: C-reactive protein
FIM: functional independence measure
FIM-C: functional independence measure-cognitive
FIM-M: functional independence measure-motor
FIM-T: functional independence measure-total
HDS-R: Hasegawa dementia rating scale-revised
J-RACT: Japanese Regimen Adherence Capacity Tests
MCC: Matthews Correlation Coefficient
RCS: Regimen comprehension scale
ROC: Receiver Operating Characteristic
